# Transcription factors CP2 and YY1 as prognostic markers in head and neck squamous cell carcinoma: analysis of The Cancer Genome Atlas and a second independent cohort

**DOI:** 10.1007/s00432-020-03482-6

**Published:** 2020-12-14

**Authors:** Julia Schnoell, Bernhard J. Jank, Lorenz Kadletz-Wanke, Stefan Stoiber, Clemens P. Spielvogel, Elisabeth Gurnhofer, Lukas Kenner, Gregor Heiduschka

**Affiliations:** 1 Department of Otorhinolaryngology, Head and Neck Surgery, Medical University of Vienna, Vienna, Austria; 2 Christian Doppler Laboratory for Applied Metabolomics, Vienna, Austria; 3 Division of Nuclear Medicine, Department of Biomedical Imaging and Image-Guided Therapy, Medical University of Vienna, Vienna, Austria; 4 Department of Pathology, Comprehensive Cancer Center, Institute of Cancer Research, Medical University of Vienna, Vienna, Austria; 5 Unit of Laboratory Animal Pathology, University of Veterinary Medicine, Vienna, Austria; 6 CBmed GmbH-Center for Biomarker Research in Medicine, Graz, Styria Austria

**Keywords:** YY1, CP2, Head and neck squamous cell carcinoma, Survival, Prognosis, Transcription factor

## Abstract

**Purpose:**

The transcription factors YY1 and CP2 have been associated with tumor promotion and suppression in various cancers. Recently, simultaneous expression of both markers was correlated with negative prognosis in cancer. The aim of this study was to explore the expression of YY1 and CP2 in head and neck squamous cell carcinoma (HNSCC) patients and their association with survival.

**Methods:**

First, we analyzed mRNA expression and copy number variations (CNVs) of *YY1* and *CP2* using “The Cancer Genome Atlas” (TCGA) with 510 HNSCC patients. Secondly, protein expression was investigated via immunohistochemistry in 102 patients, who were treated in the Vienna General Hospital, utilizing a tissue microarray.

**Results:**

The median follow-up was 2.9 years (1.8–4.6) for the TCGA cohort and 10.3 years (6.5–12.8) for the inhouse tissue micro-array (TMA) cohort. The median overall survival of the TCGA cohort was decreased for patients with a high *YY1* mRNA expression (4.0 vs. 5.7 years, *p* = 0.030, corr. *p* = 0.180) and high *YY1*-CNV (3.53 vs. 5.4 years, *p* = 0.0355, corr. *p* = 0.213). Furthermore, patients with a combined high expression of *YY1* and *CP2* mRNA showed a worse survival (3.5 vs. 5.4 years, *p* = 0.003, corr. *p* = 0.018). The mortality rate of patients with co-expression of *YY1* and *CP2* mRNA was twice as high compared to patients with low expression of one or both (HR 1.99, 95% CI 1.11–3.58, *p* = 0.021). Protein expression of nuclear YY1 and CP2 showed no association with disease outcome in our inhouse cohort.

**Conclusion:**

Our data indicate that simultaneous expression of *YY1* and *CP2* mRNA is associated with shorter overall survival. Thus, combined high mRNA expression might be a suitable prognostic marker for risk stratification in HNSCC patients. However, since we could not validate this finding at genomic or protein level, we hypothesize that unknown underlying mechanisms which regulate mRNA transcription of YY1 and CP2 are the actual culprits leading to a worse survival.

**Supplementary Information:**

The online version contains supplementary material available at 10.1007/s00432-020-03482-6.

## Introduction

Head and neck squamous cell carcinoma (HNSCC) is the sixth most frequent cancer worldwide and about 650,000 new cases are diagnosed each year (Cognetti et al. 2008). The reported 5-year survival rates range between 25 and 59% and strongly depend on the anatomic site and stage at presentation (Gatta et al. 2015). Treatment options include surgical resection, radio- and chemotherapy. Although therapy has evolved and survival rates have improved, about one-third of patients die within 5-years of diagnosis (Pulte and Brenner 2010). To date, known risk factors for poor survival are smoking, poor socioeconomic status and a negative human papilloma virus (HPV) status in oropharyngeal SCC patients (Gatta et al. 2015). While treatment de-escalation is currently under clinical investigation in HPV-positive oropharyngeal SCC patients, biomarkers for better therapeutic decision guidance in HNSCC are still lacking (Mirghani and Blanchard 2018).

The transcription factors Ying Yang 1 (YY1) and CP2 have been shown to act as tumor suppressors and promoters. YY1 is an ubiquitous transcription activator and repressor and is involved in the transcriptional regulation of approximately 10% of the human exome (Gordon et al. 2006; Khachigian 2018). Among many different functions, it is involved in the regulation of cell proliferation, apoptosis, DNA repair, chromatin modelling and epigenetic modification (Sarvagalla et al. 2019). While YY1 acts as a tumor suppressor in pancreatic cancer (Zhang et al. 2016), it exhibits a tumor promoting function in colon cancer (Yokoyama et al. 2010) and prostate cancer (Camacho-Moctezuma et al. 2018).

The transcription factor CP2, also known as Late SV40 Factor (LSF) and LBP-1c, is ubiquitously expressed as well. It is involved in hematopoiesis, regulation of the cell cycle and expression of immune-related genes. CP2 is overexpressed in many cancers, such as hepatocellular carcinoma (Yoo et al. 2010), pancreatic cancer (Yuedi et al. 2017) and colorectal cancer (Jiang et al. 2014), and mostly serves as a pro-oncogene (Kotarba et al. 2018). However, in melanoma cells, overexpression of CP2 leads to growth inhibition (Goto et al. 2016).

One possible explanation for the controversial effects in tumors of both transcription factors is the interaction with other proteins. In hepatocellular carcinoma, the co-expression of the transcription factors YY1 and CP2 was associated with a significantly worse prognosis (Kim et al. 2017). This was the first report that proposed the joint expression of both genes as prognostic markers in a tumor. In fact, data on the interaction of YY1 and CP2 are sparse. Structural and interaction analysis showed that YY1 is able to bind to CP2 (Coull et al. 2000; Kang et al. 2005) and together they are involved in spermatogenesis (Kim et al. 2016) and HIV replication (Romerio et al. 1997; Coull et al. 2000).

Hence, in this study, we investigated the mRNA expression and copy number variation (CNV) of *YY1* and *CP2* and their individual and combined prognostic relevance in HNSCC. Furthermore, we analyzed their expression at the protein level in an independent patient cohort.

## Patients and methods

### The Cancer Genome Atlas dataset

Data from “The Cancer Genome Atlas” (TCGA), namely “TCGA, Firehose Legacy” including 530 samples were extracted from cBioportal.org. Missing data were extracted from “TCGA PanCancer Atlas” including 523 samples (Liu et al. 2018), and “TCGA, Nature 2015” including 279 samples (Lawrence et al. 2015). Patients with incomplete survival data or missing mRNA data were excluded as well as patients with an overall survival (OS) of less than 2 months due to the possibility of perioperative complications (*n* = 11). HPV status was assessed via RNA sequencing as described by Liu et al. (2018, Suppl. Information). Most patients received surgical resection with curative intent and postoperative radiotherapy (Liu et al. 2018, Suppl. Information). However, treatment options also included neoadjuvant radiotherapy or pharmaceutical therapy. Only few patients received primary radiotherapy and/or chemotherapy (see Table 1). mRNA expression *z* scores (RNA Seq V2 RSEM) were extracted for *YY1* and *TFCP2* via cBioportal.org. Thus, after merging these datasets we were able to include 510 patients in total. mRNA expression was divided into low and high expression with a cutoff at a *z* score of > 0 for high mRNA expression. CNV data were retrieved from UCSC Xena for *YY1* and *TFCP2* (Goldman et al. 2020). CNV calculation was performed as described by the copy number variation analysis pipeline of the National Cancer Institute GDC Documentation (Copy Number Variation Analysis Pipeline). The cohort was divided by the median copy number.

**Table 1:** Basic data and descriptive statistics of the primary (TCGA) and secondary (TMA) dataset of HNSCC patients

	Primary dataset (TCGA)			Secondary dataset (TMA)	
	Total (*n* = 510)	Percent (%)	chi^2^ *p* value	Total (*n* = 102)	Percent (%)
Sex					
Female	132	26		23	23
Male	378	74	0.480	79	77
Age					
< 60	231	45		41	40
≥ 60	279	55	0.344	61	60
Primary					
Oral cavity	307	60		20	20
Oropharynx	79	15		53	52
Hypopharynx	10	2		18	18
Larynx	114	22	**< 0.001**	11	11
HPV					
Negative	413	81		74	7
positive	76	15	**0.019**	25	25
x	21	4		3	3
T stage					
1	34	7		21	21
2	148	29		52	51
3	133	26		19	19
4	180	35	**< 0.001**	10	10
x	15	3		0	0
N stage					
0	237	46		7	7
1	81	16		29	28
2	162	32		63	62
3	9	2	**< 0.001**	3	3
x	21	4		0	0
M stage					
0	480	94		70	69
1	6	1	0.350	0	0
x	24	5		32	31
Staging					
I	20	4		1	1
II	94	18		3	3
III	103	20		27	26
IV	280	55	**< 0.001**	71	70
x	13	3		0	0
Smoker					
Never/ex	323	63		40	39
Active	173	34	**< 0.001**	62	61
x	14	3		0	0
Radiotherapy					
No	86	17		0	0
Adjuvant	189	37		102	100
Primary	18	4		0	0
Neoadjuvant	6	1	**< 0.001**	0	0
x	211	41		0	0
Pharmaceutical therapy					
No	129	25		85	83
Adjuvant	113	22		16	16
Primary	4	1		0	0
Neoadjuvant	8	2	**< 0.001**	0	0
x	256	50		0	0

*p* value below 0.05 was considered significant and highlighted (bold)

Statistical correlations to the TMA dataset were analyzed using Chi-squared test. A

### Tissue microarray patient dataset

In this cohort, we included 102 HNSCC patients from a single center, retrospective study at the Vienna General Hospital. Patients received surgery, postoperative radiotherapy and additional chemotherapy in case of extranodal spread between 2002 and 2012. Patients were excluded when they had a second primary carcinoma, received external treatment, showed distant metastasis, had prior irradiation or were under immunosuppression. Data were collected by medical chart review. HPV status was assessed using in situ hybridization. Collected data included date of birth, time of initial diagnosis, recurrence, tumor grading, histology and date of death or date of last follow-up. This study was approved by the ethics committee of the Medical University of Vienna (EK1262/2019).

### Tissue microarray

Tissue samples were taken from preselected formalin-fixed, paraffin-embedded (FFPE) HNSCC tissue acquired through surgical resection. The tissue microarray (TMA) was constructed using a Galileo TMA CK Series-HTS Tissue computer assisted TMA Microarray Platform (Integrated Systems Engineering Srl, Milan, Italy). Histology was confirmed by hematoxylin–eosin (H&E) staining. Subsequently, 4 µm sections were prepared for immunohistochemical analysis.

### Immunohistochemistry

Immunohistochemical staining was performed using a Lab Vision Ultra Kit (Thermo Scientific) according to the manufacturer’s protocol. In short, the appropriate retrieval buffer and antibody dilution were assessed prior to analysis using colon and stomach tissue as positive control. After dewaxing and dehydrating the TMA, antigen retrieval was performed using EDTA in a microwave oven at 600 W for 10 min. Endogenous peroxidase activity was blocked in 3% H_2_O_2_ and Ultra V Block was applied. Subsequently, the tissue was incubated for 1 h with the primary antibodies against YY1 1:100 (sc-7341, Santa Cruz Biotechnology) and CP2 1:200 (610818, BD Biosciences) at room temperature. Then, the primary antibody enhancer and horseradish peroxidase enhancer were applied for 10 and 15 min, respectively. UltraVision Plus Detection System DAB Plus Substrate System (Thermo Scientific) was used to visualize staining. Tissues were counterstained with hematoxylin Gill II (Merck). The tissues were scanned using an NIKON Eclipse Ti microscope (NIKON) and analyzed using a modified ImageJ plugin of “IHC Profiler” (Varghese et al. 2014). Since the original plugin uses threshold adjustment and selection in the deconvoluted DAB image to select nuclei or tissue, all nuclei or tissue with negative staining were not considered. Therefore, the script was changed to adjust the threshold in the binarized original image before deconvolution to include either all nuclei or the whole tissue area. The threshold was then used for selection to analyze nuclear YY1 or overall CP2 DAB intensity in the deconvoluted image. Expression levels were categorized by the IHC Profiler under visual control of the researcher (J.S.) in negative, low positive, positive, and high positive expression levels. Negative and low positive expression levels were further grouped into low expression, and positive and high positive expression were considered as high expression.

### Statistical analysis

To further analyze the combined expression of YY1 and CP2, the YY1CP2 score was formed. The YY1CP2 score was considered positive when mRNA, CNV or protein levels of both YY1 and CP2 were high (high YY1/high CP2). When both levels were low, or only one was high, the YY1CP2 score was considered negative (low YY1/low CP2, low YY1/high CP2, high YY1/low CP2). Categorical data were reported as absolute frequencies (%) and continuous data as median as well as 25th and 75th percentiles. OS or disease-free survival (DFS) were calculated from the date of diagnosis to the date of death or tumor recurrence, respectively. Kaplan–Meier curves were calculated to visualize OS and DFS rates and analyzed for statistical significance using log-rank test. Uni- and multivariable regression analysis was calculated using the Cox proportional hazard model. The multivariable model was corrected for staging, HPV, and smoker status. A pairwise-interaction analysis between *YY1* and *CP2* mRNA expression was performed to further analyze the impact of combined high expression on OS. The median follow-up was calculated using the method published by Schemper and Smith (1996). Statistical analysis was performed using Prism GraphPad software (GraphPad Software, Inc, La Jolla, CA) and Stata (Stata Corp, Houston, TX).

## Results

### Analysis at baseline

Five hundred and ten (510) patients were included in the TCGA dataset. Patient characteristics are shown in Table 1. The median observation period was 2.9 years (1.8–4.6). The median age at diagnosis was 60.5 years (53–68). The 5-year OS was 41% and the 5-year DFS was 45%. Treatment options included surgery, radiotherapy and pharmaceutical therapy as specified in Table 1; however, it is unspecified in up to 50%.

### Analysis of expression of *YY1* and *CP2* mRNA and clinicopathological data

mRNA levels were split into high or low levels with a cutoff at a *z* score of 0. *YY1* mRNA levels were high in 54% (275) and *CP2* mRNA levels were high in 50% (253) of the samples. Since mRNA levels of *YY1* and *CP2* showed a low positive correlation (Fisher’s exact *p* = 0.027, correlation coeff. *r* = 0.221) we formed the *YY1CP2* score. Twenty-nine percent (149) showed a positive *YY1CP2* score (high expression of *YY1* and *CP2*).

The expression of *YY1*, *CP2* and the *YY1CP2* score was analyzed for its association with various clinicopathological features (Suppl. Table 1). Low mRNA expression of *CP2* was more commonly found in patients with a higher T stage (T3–T4; *p* = 0.005, corr. *p* = 0.030) and HPV-negative patients (*p* = 0.002, corr. *p* = 0.012). Furthermore, male patients more commonly showed high *YY1* mRNA expression (*p* = 0.008, corr. *p* = 0.048). After Bonferroni correction, there was no further significant correlation. The *YY1CP2* score showed no association with clinicopathological data.

### Analysis of expression of *YY1* and *CP2* copy number variation and clinicopathological data

CNVs were divided into high or low using the median as cutoff. *YY1*-CNVs were high in 50% (254) and *CP2*-CNVs were high in 28% (143) of the samples. The *YY1CP2*- score was high in 28% (143). While the categorical evaluation of *YY1*- and *CP2*-CNVs showed a correlation, the continuous CNVs showed only a very weak correlation (Fisher’s exact *p* = 0.002, correlation coeff. *r* = − 0.076).

The correlation of CNV to clinicopathological data revealed an association of *YY1*-CNV with HPV (*p* < 0.001, corr. *p* < 0.006) and smoker status (*p* = 0.006, corr. *p* = 0.036). There was no further significant correlation of *YY1*-, *CP2*-CNV or the CNV-*YY1CP2* score after Bonferroni correction.

We then analyzed whether there was a correlation of mRNA and CNV values. *YY1* mRNA showed a strong correlation with CNV values (Fisher’s exact *p* < 0.001; correlation coeff. *r* = 0.618). *CP2* mRNA expression revealed a moderate correlation with CNV (Fisher’s exact *p* < 0.001; correlation coeff. *r* = 0.441).

### Analysis of overall survival and disease-free survival

**Fig. 1:**
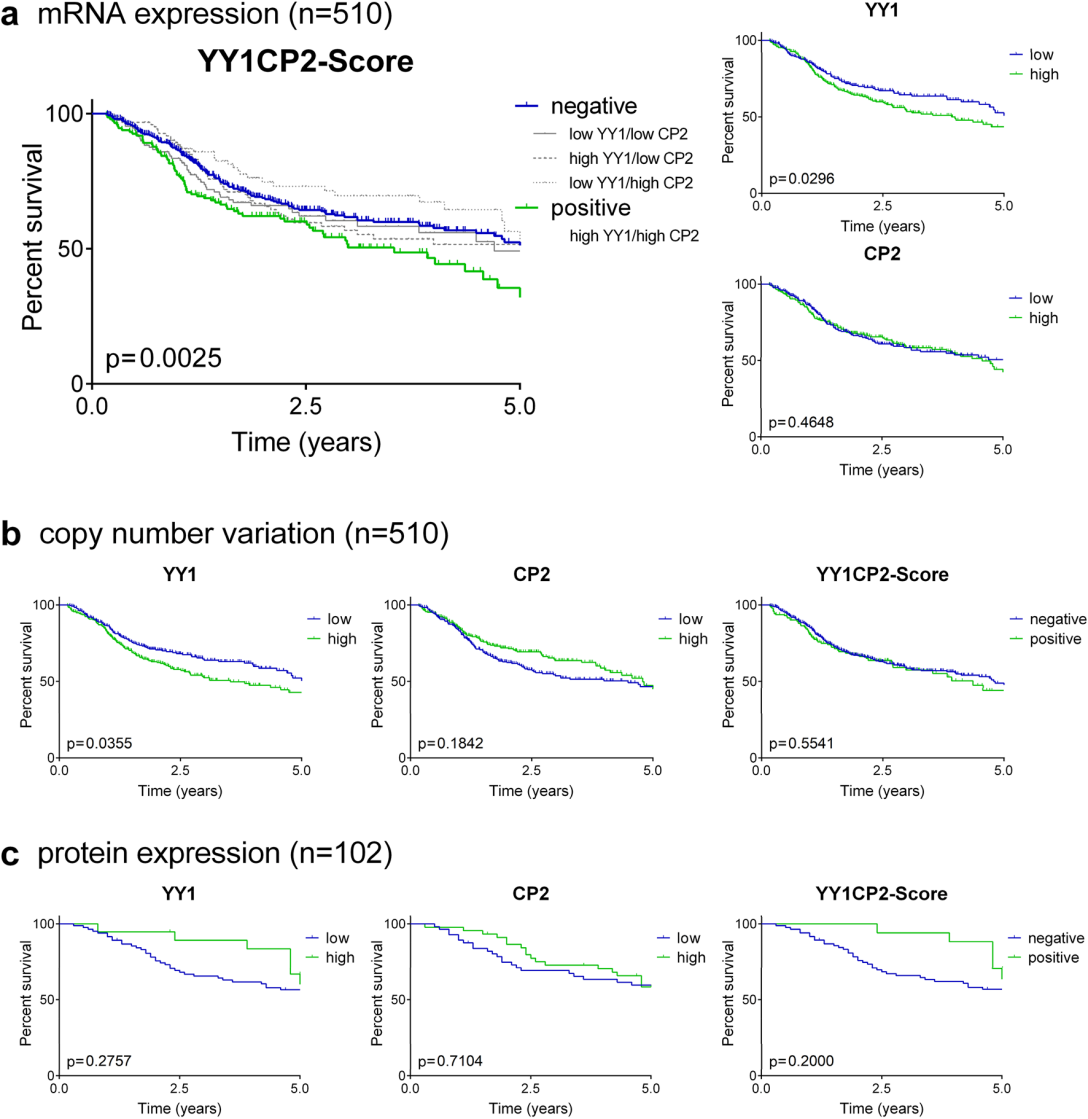
Kaplan–Meier curves of overall survival for the **a** mRNA expression, **b** CNV and **c** protein expression of YY1, CP2 and the YY1CP2 score. Survival was analyzed using log-rank test. *p*, log-rank *p* value

To determine whether levels of *YY1* or *CP2* mRNA expression or CNV might be associated with disease outcome in HNSCC patients, we examined OS and DFS in the TCGA. As shown in Fig. 1, survival was decreased for patients with high expression of *YY1* (4.0 vs. 5.7 years, *p* = 0.030, corr. *p* = 0.180). Furthermore, the median OS was decreased in case of a positive *YY1CP2* score (3.5 vs. 5.4 years, *p* = 0.003, corr. *p* = 0.018). When CNV data were analyzed, only patients with a high *YY1*-CNV showed a worse survival (3.5 vs. 5.4 years, *p* = 0.0355, corr. *p* = 0.213). There was no further association of mRNA levels, CNV or (CNV-)*YY1CP2* score with OS or DFS. Kaplan–Meier curves for DFS were calculated as shown in Suppl. Figure 1.

Univariable analysis showed a worse OS for patients with high *YY1* mRNA levels (HR 1.35, 95% CI 1.03–1.78, *p* = 0.030, corr. *p* = 0.180). This result did not prevail in multivariable analysis after correction for stage, smoker status and HPV status (Table 2). Further analysis of the risk per increase of *z* score unit did not reach statistical significance. Univariable analysis of *CP2* expression or *z* score did not show a significant association with outcome. However, after correction for multiple confounders, patients showed an increased risk for death of 14% per increase in *z* score (HR 1.14, 95% CI 1.00–1.29, *p* = 0.049, corr. *p* = 0.294). Patients with a positive *YY1CP2* score showed a significantly increased risk for death in uni- (HR 1.55, 95% CI 1.16–2.05, *p* = 0.003, corr. *p* = 0.018) and multivariable analysis (HR 1.60, 95% CI 1.19–2.16, *p* = 0.002, corr. *p* = 0.012). To determine whether those two mRNA markers significantly interact, we added pairwise-interaction terms to the cox regression model. This revealed no individual increase in risk for high mRNA expression of only one (*YY1* or *CP2*). However, when both mRNA expression levels were high, uni- and multivariable pairwise-interaction analysis revealed a significant association with a worse OS (univariable: HR 1.96, 95% CI 1.12–3.43, *p* = 0.018; multivariable: HR 1.99, 95% CI 1.11–3.58, *p* = 0.021).

**Table 2:** Uni- and multivariable analysis of overall survival for mRNA expression, CNV and protein expression of YY1, CP2 and the YY1CP2 score

	Univariable			Multivariable		
	HR	95% CI	*p* value	HR	95% CI	*p* value
mRNA expression (*n* = 510)						
YY1 high vs. low	1.35	1.03–1.78	**0.030**	1.26	0.94–1.68	0.127
YY1 *z* score	1.08	0.99–1.19	0.068	1.05	0.94–1.18	0.365
CP2 high vs. low	1.11	0.84–1.45	0.465	1.24	0.94–1.65	0.133
CP2 *z* score	1.07	0.95–1.20	0.262	1.14	1.00–1.29	**0.049**
YY1CP2 score	1.55	1.16–2.05	**0.003**	1.60	1.19–2.16	**0.002**
mRNA expression—interaction analysis (*n* = 510)						
YY1 + (ref. YY1 − CP2 −)	0.97	0.66–1.43	0.892	0.89	0.59–1.34	0.570
CP2 + (ref. YY1 − CP2 −)	0.74	0.48–1.13	0.165	0.82	0.52–1.29	0.398
YY1 + CP2 +	1.96	1.12–3.43	**0.018**	1.99	1.11–3.58	**0.021**
CNV (*n* = 510)						
YY1 high vs. low	1.33	1.02–1.75	**0.036**	1.19	0.89–1.59	0.230
YY1 values	1.20	0.63–2.31	0.582	1.10	0.57–2.12	0.778
CP2 high vs. low	0.83	0.63–1.09	0.185	0.90	0.68–1.20	0.479
CP2 values	0.88	0.30–2.58	0.813	1.16	0.37–3.58	0.803
YY1CP2 score	1.10	0.81–1.49	0.555	1.07	0.78–1.48	0.669
Protein expression (*n* = 102)						
YY1	0.66	0.31–1.40	0.281	0.63	0.28–1.44	0.275
CP2	0.90	0.52–1.57	0.712	1.05	0.59–1.88	0.861
YY1CP2 score	0.60	0.27–1.33	0.207	0.66	0.29–1.51	0.320

*p* value below 0.05 was considered significant and highlighted (bold)

mRNA levels were analyzed for their expression level (high vs. low) and per change of *z* score unit. Multivariable analysis was adjusted for staging, HPV status and smoker status. For mRNA expression, the pair-wise interaction for high expressions of *YY1* and *CP2* was calculated. A *p* value below 0.05 was considered significant and highlighted (bold).

*HR*, hazard ratio, *CI* confidence interval, *ref.* reference, + high expression, − low expression

Analysis of CNV data revealed a higher risk for death in case of high *YY1*-CNV in univariable analysis (HR 1.33, 95% CI 1.02–1.75, *p* = 0.036, corr. *p* = 0.216). However, this result did not prevail in multivariable analysis, and uni- and multivariable analysis of continuous CNV values. Furthermore, analysis of *CP2*-CNV or the calculated CNV-*YY1CP2* score did not show an association with outcome.

To better compare the two datasets, we analyzed only TCGA patients who received postoperative radio(chemo)therapy. *YY1* and *CP2 mRNA* were not associated with OS. However, patients with a combined high expression of *YY1* and *CP2* showed a significantly shorter OS (3.9 vs. 5.7 years, log-rank *p* = 0.038, corr. *p* = 0.114). Further analysis of the *YY1CP2* score revealed an increased risk for death (HR 1.69, 95% CI 1.02–2.79, *p* = 0.040, corr. *p* = 0.120) independent of multiple confounders (HR 1.73, 95% CI 1.04–2.87, *p* = 0.034, corr. *p* = 0.102). Interaction analysis further confirmed the association with OS (univariable: HR 3.67, 95% CI 1.27–10.60, *p* = 0.016; multivariable: HR 3.49, 95% CI 1.19–10.19, *p* = 0.023).

### Tissue microarray dataset

**Fig. 2:**
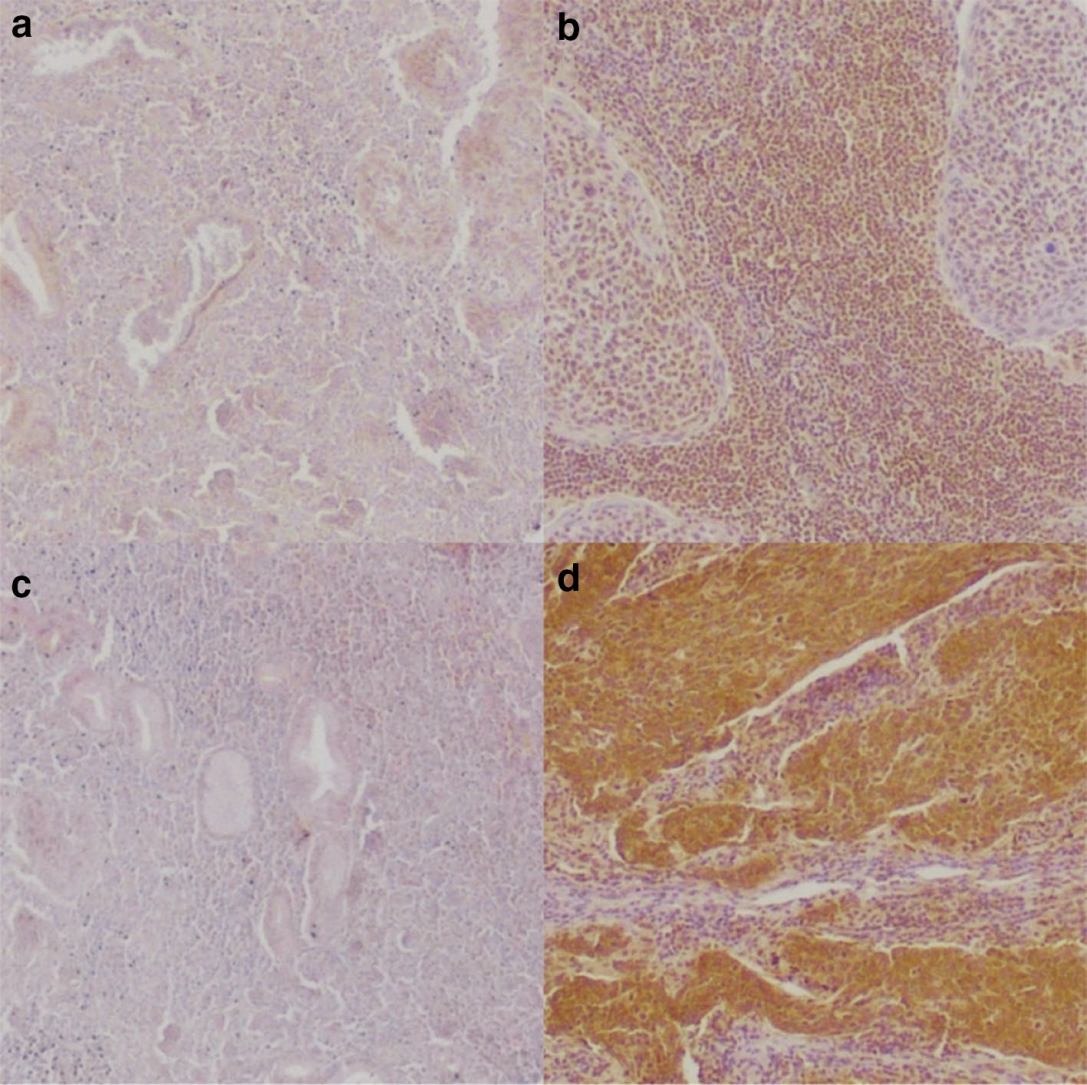
Images of protein expression of immunohistochemical staining of **a** low and **b** high nuclear YY1, and **c** low and **d** high CP2

To further explore the association of YY1 and CP2 with disease outcome, we examined expression at the protein level using immunohistochemistry (Fig. 2). In total, 102 patients with HNSCC were included in our secondary dataset. Patient characteristics are presented in Table 1. The median observation period was 10.3 years (6.5–12.8). The median age at diagnosis was 59 (53–63). The 5-year OS was 56% and the 5-year DFS was 67%. All patients received postoperative radiotherapy with a median dose of 60 Gy. Sixteen percent (16) received additional chemotherapy due to extracapsular spread or R1 resection.

Immunohistochemical staining showed high expression of nuclear YY1 in 19% (19) and high expression of CP2 in 44% (45). High expression of YY1 showed a moderate positive correlation with high expression of CP2 (Fisher’s exact *p* < 0.001, correlation coeff. *r* = 0.491). After combining the expressions of YY1 and CP2 to form the YY1CP2 score (as described earlier), 17% (17) showed a positive score.

Next, we tested for associations of marker expression with clinicopathological characteristics. Low YY1 protein expression (*p* = 0.014, corr. *p* = 0.042) and a negative YY1CP2 score (*p* = 0.011, corr. *p* = 0.033) were associated with a negative HPV status (Suppl. Table 2). There was no further association of protein expression or YY1CP2 score with other clinicopathological features.

Finally, we analyzed marker expression at the protein level and its association with disease outcome. No significant association of protein expression or the YY1CP2 score with OS or DFS was observed (Table 2, Suppl. Table 3). Kaplan–Meier curves for OS and DFS were calculated as shown in Fig. 1 and Suppl. Figure 1, respectively.

### Statistical comparison of the two cohorts

As seen in Table 1, the two cohorts show differences in the locations of the primary. The TCGA dataset generally contains more locally advanced stages (*p* < 0.001) with a lower N stage (*p* < 0.001). Resulting in more early-staged cancers (I–II, *p* < 0.001). Interestingly, the TCGA cohort shows a generally worse OS (4.7 vs 7.1 years, *p* = 0.003) and DFS (4.7 vs. 13 years, *p* < 0.001). Observation time differs between the cohorts (2.9 vs. 10.3 years, *p* < 0.001). The TCGA cohort contains fewer active smokers (*p* < 0.001) and more patients with an HPV negative status (*p* = 0.019), probably due to less patients with oropharyngeal carcinoma. Patients within the TCGA cohort are older (61 vs. 58 years, *t* test *p* = 0.028, chi^2^ *p* = 0.344). Furthermore, the application of radiotherapy (*p* < 0.001) and chemotherapy (*p* < 0.001) differs greatly; however, for up to 50% of the patient’s therapy is not further specified in the TCGA cohort. For better comparison to the TMA cohort, we analyzed the TCGA subgroup who received postoperative radio(chemo)therapy. However, this subgroup still shows significant differences in the location of the primary, T stage, N stage, smoker status and the application of pharmaceutical therapy (data not shown, all *p* < 0.001).

## Discussion

HNSCC is among the ten most frequently diagnosed malignancies worldwide. Survival has improved over the last 2 decades, still about one-third of HNSCC patients die within 5 years of diagnosis (Pulte and Brenner 2010). If these high-risk patients could be identified early in the course of their disease, intensified treatment could potentially be beneficial. Hence, it is necessary to find prognostic markers to stratify high-risk patients.

The transcription factors YY1 and CP2 act as tumor suppressors and promoters. A combined high protein expression of these factors was recently linked to a worse prognosis in hepatocellular carcinoma (Kim et al. 2017). To date, there is only little data on the role of these transcription factors in HNSCC. Noteworthy, YY1 protein expression is associated with enhanced proliferation and migration in oral cancer cells (Behera et al. 2019). Furthermore, high expression of YY1 mRNA and protein was associated with pro-neoplastic effects in laryngeal cancer (Qu et al. 2017). For CP2 protein expression, a report in oral carcinoma showed an upregulation and association with higher tumor and TNM stage (Chen et al. 2017). The purpose of this study was to investigate the individual role of YY1 and CP2 in HNSCC as well as a statistical add on effect of their co-expression.

First, we evaluated the mRNA expression levels of *YY1* and *CP2* in a cohort of HNSCC patients, which we extracted from “The Cancer Genome Atlas, Firehose Legacy”. Although Kaplan–Meier curves revealed a trend for worse survival of patients with a high *YY1* or *CP2* mRNA expression, further statistical analysis was controversial. High *YY1* mRNA expression was associated with a shorter median survival in univariable analysis but did not prevail after correcting for multiple confounders. High expression of *CP2* mRNA was not associated with survival. Further analysis of the association of survival with continuous *z* scores revealed no significant influence of the *YY1* *z* score. Interestingly, a higher *CP2* *z* score showed an increased risk for death after multivariable correction. In literature, *YY1* mRNA expression was not associated with survival in cancers of the central nervous system, the breast, the colon, and the lungs (Bonavida and Kaufhold 2015). In contrast, high expression of *YY1* mRNA was associated with a worse outcome in diffuse large B-cell lymphoma (DLBCL) (Sakhinia et al. 2007) but showed a better outcome in pancreatic cancer (Zhang et al. 2014). In hepatocellular carcinoma, high expression of *CP2* mRNA was associated with a worse DFS but not with OS (Kim et al. 2017).

We subsequently formed a *YY1CP2* score to compare patients with simultaneous high levels of *YY1* and *CP2* mRNA (positive *YY1CP2* score) to the group with a negative *YY1CP2* score. Patients with a positive *YY1CP2* score showed a shorter median survival and this turned out to be an independent prognostic marker for worse OS in multivariable analysis. We then performed an interaction analysis and found that the combined high *YY1* and *CP2* status proved to be an independent prognostic marker for worse OS. In contrast, no association with prognosis was found for individual high expression of either *YY1* or *CP2* in this interaction model. Interestingly, analysis of DFS showed no association with *YY1*, *CP2* or the *YY1CP2* score. Moreover, there was no association of the *YY1CP2* score with clinicopathological features, suggesting its independence from potential confounders. In accordance with our findings, in hepatocellular carcinoma, survival was decreased in case of combined high protein expression of *CP2* and *YY1* (Kim et al. 2017).

Next, we analyzed CNVs of *YY1* and *CP2*. In accordance with our findings at mRNA level, patients with a high *YY1*-CNV showed a shorter OS. However, this result did not prevail after correction for multiple confounders. Likewise, there was no other association of *YY1*- or *CP2*-CNV with DFS or OS. Although mRNA and CNV values showed a moderate-to-strong positive correlation, the analysis of the formed CNV-*YY1CP2* score showed no association with OS or DFS. In contrast to our findings, *CP2*-CNV was associated with DFS in hepatocellular carcinoma (Kim et al. 2017).

To verify our findings at the protein level, we investigated the expression of CP2 and nuclear YY1 in a secondary dataset using immunohistochemistry. In concordance with our findings on mRNA level, expression of CP2 and nuclear YY1 showed a positive correlation. However, we could not find a significant association with OS or DFS. Likewise, Kim et al. found no significant association of CP2 or YY1 expression with OS in hepatocellular carcinoma; however, CP2 expression was associated with a worse DFS (Kim et al. 2017). In contrast, Jiang et al. found that high CP2 protein expression was associated with a worse prognosis in hepatocellular carcinoma (Jiang et al. 2014). For YY1, high expression was associated with a worse outcome in hepatoblastoma (Shin et al. 2011), DLBCL and follicular lymphoma (Sakhinia et al. 2007). Interestingly, high expression of YY1 was also associated with a longer DFS in prostate cancer (Seligson et al. 2005) and a better outcome in pancreatic cancer (Zhang et al. 2014), colon cancer (Chinnappan et al. 2009) and follicular lymphoma (Naidoo et al. 2011).

Altogether, the question remains whether the transcription factors YY1 and CP2 influence survival via joint activation or suppression of other genes (our data suggest otherwise), or unknown underlying mechanisms which regulate mRNA transcription of YY1 and CP2 are the actual culprits leading to a worse outcome. In the literature, *YY1* and *CP2* mRNA expression generally correlated with protein expression (Jiang et al. 2014; Zhang et al. 2014). A possible explanation for our divergent results of mRNA and protein expression is the influence of posttranscriptional processes to protein abundance (Liu et al. 2016). Furthermore, there might be some selection bias since our secondary dataset only included tissue from patients who received surgical therapy and postoperative radio(chemo)therapy. Thus, we performed a short analysis of the TCGA subgroup who received postoperative radio(chemo)therapy. The association of combined high mRNA expression of *YY1* and *CP2 with OS* did not change significantly; however, the subgroup still showed differences in patient characteristics. All in all, there was no relevant correlation of mRNA or protein expression with clinicopathological data, which would explain the different results of the two cohorts. This study design, however, is not suitable to evaluate the relationship between mRNA and protein levels of YY1 and CP2 since they were investigated in different patient cohorts.

Limitations of this study are the comparison of mRNA expression with protein expression in different cohorts. Although protein levels largely depend on mRNA levels, this is a complicated relationship and can easily vary through post-transcriptional processes, protein half-lives and the error or noise of the experiments (Liu et al. 2016). Both datasets are limited due to their retrospective designs. Furthermore, the secondary dataset shows a considerably smaller number of patients. Protein expression was investigated in FFPE tissue; thus, some protein might be lost compared with fresh tissue. Furthermore, staining intensity was evaluated using a modification of the ImageJ plugin of “IHC Profiler” (Varghese et al. 2014) under visual control of one fully blinded researcher (J.S.). All in all, external validation of our findings at the mRNA, CNV and protein level are necessary.

In conclusion, we showed for the first time that high co-expression of *YY1* and *CP2* mRNA is an independent prognostic marker for a worse OS in HNSCC. However, we were not able to validate these findings at the protein level in an independent cohort. Nonetheless, we hypothesize that our data show a prognostic relevance of combined high expression of *YY1* and *CP2* mRNA in HNSCC and this warrants further investigation.

## Supplementary Information

Below is the link to the electronic supplementary material.

Supplementary file1 (DOCX 54 KB)

## Data Availability

The datasets generated during and/or analyzed during the current study are available from the corresponding author on reasonable request.

## Abbreviations

CNV: Copy number variation
DFS: Disease-free survival
DLBCL: Diffuse large B cell lymphoma
FFPE: Formalin-fixed, paraffin-embedded
HNSCC: Head and neck squamous cell carcinoma
LSF: Late SV40 Factor
OS: Overall survival
SCC: Squamous cell carcinoma
TCGA: The Cancer Genome Atlas
TMA: Tissue microarray
YY1: Ying Yang 1
