# Supplementary material for: Transcription factors CP2 and YY1 as prognostic markers in head and neck squamous cell carcinoma: analysis of The Cancer Genome Atlas and a second independent cohort
Source: J Cancer Res Clin Oncol. 2020 Dec 14;147(3):755–65. doi: 10.1007/s00432-020-03482-6 (PMC7872999; doi:10.1007/s00432-020-03482-6)
Supplement: Supplementary file 1 — Supplementary file1 (DOCX 54 KB) [file 432_2020_3482_MOESM1_ESM.docx]

Supplementary Table 1 Correlation analysis for associations between mRNA expression and CNV of *YY1*, *CP2* and the *YY1CP2*-score and clinicopathological features. Correlations were analyzed using Fisher’s exact test or Chi-squared test. HPVhr, HPV high risk

|  |  | **mRNA expression (n=510)** | | | | | | **Copy number variation (n=510)** | | | | | |
| --- | --- | --- | --- | --- | --- | --- | --- | --- | --- | --- | --- | --- | --- |
|  |  | **YY1** | | **CP2** | | **YY1CP2** | | **YY1** | | **CP2** | | **YY1CP2** | |
|  |  | **low** | **high** | **low** | **high** | **negative** | **positive** | **low** | **high** | **low** | **high** | **negative** | **positive** |
| **T Stage** | **T1-2** | 94 (41%) | 88 (33%) | 77 (31%) | 105 (43%) | 128 (36%) | 54 (38%) | 101 (41%) | 81 (33%) | 92 (51%) | 160 (51%) | 133 (73%) | 226 (72%) |
|  | **T3-4** | 134 (59%) | 179 (67%) | 173 (69%) | 140 (57%) | 223 (64%) | 90 (63%) | 148 (59%) | 165 (67%) | 90 (49%) | 153 (49%) | 49 (27%) | 87 (28%) |
|  |  | p=0.057 | | **p=0.005** | | p=0.829 | | p=0.093 | | p=0.926 | | p=0.917 | |
| **N Stage** | **N0** | 112 (50%) | 125 (47%) | 112 (46%) | 125 (51%) | 161 (47%) | 76 (52%) | 115 (47%) | 122 (50%) | 122 (49%) | 115 (48%) | 164 (46%) | 73 (54%) |
|  | **N1-3** | 111 (50%) | 141 (53%) | 134 (54%) | 118 (49%) | 183 (53%) | 69 (48%) | 130 (53%) | 122 (50%) | 128 (51%) | 124 (52%) | 191 (54%) | 61 (46%) |
|  |  | p=0.476 | | p=0.191 | | p=0.257 | | p=0.527 | | p=0.928 | | p=0.106 | |
| **Staging** | **I-II** | 59 (26%) | 55 (20%) | 50 (20%) | 64 (26%) | 79 (22%) | 35 (24%) | 62 (25%) | 52 (21%) | 62 (24%) | 52 (21%) | 82 (23%) | 32 (24%) |
|  | **III-IV** | 169 (74%) | 214 (80%) | 201 (80%) | 182 (74%) | 273 (78%) | 110 (76%) | 189 (75%) | 194 (79%) | 192 (76%) | 191 (79%) | 279 (77%) | 104 (76%) |
|  |  | p=0.151 | | p=0.106 | | p=0.683 | | p=0.393 | | p=0.456 | | p=0.905 | |
| **HPVhr** | **negative** | 189 (83%) | 224 (86%) | 223 (90%) | 190 (79%) | 297 (85%) | 116 (84%) | 190 (78%) | 223 (91%) | 218 (88%) | 195 (81%) | 292 (83%) | 121 (88%) |
|  | **positive** | 40 (17%) | 36 (14%) | 26 (10%) | 50 (21%) | 54 (15%) | 22 (16%) | 54 (22%) | 22 (9%) | 30 (12%) | 46 (19%) | 60 (17%) | 16 (12%) |
|  |  | p=0.270 | | **p=0.002** | | p=0.878 | | **p<0.001** | | p=0.035 | | p=0.165 | |
| **Sex** | **female** | 74 (31%) | 58 (21%) | 63 (25%) | 69 (27%) | 92 (25%) | 40 (27%) | 67 (26%) | 65 (26%) | 71 (27%) | 61 (24%) | 95 (26%) | 37 (26%) |
|  | **male** | 161 (69%) | 217 (79%) | 194 (75%) | 184 (73%) | 269 (75%) | 109 (73%) | 189 (74%) | 189 (74%) | 188 (73%) | 190 (76%) | 272 (74%) | 106 (74%) |
|  |  | **p=0.008** | | p=0.477 | | p=0.750 | | p=0.920 | | p=0.479 | | p=1.000 | |
| **Age** | **<60** | 100 (43%) | 131 (48%) | 123 (45%) | 108 (43%) | 169 (47%) | 62 (42%) | 114 (45%) | 117 (46%) | 123 (47%) | 108 (43%) | 174 (47%) | 57 (40%) |
|  | **≥60** | 135 (57%) | 144 (52%) | 134 (52%) | 145 (57%) | 192 (53%) | 87 (58%) | 142 (55%) | 137 (54%) | 136 (53%) | 143 (57%) | 193 (53%) | 86 (60%) |
|  |  | p=0.250 | | p=0.241 | | p=0.283 | | p=0.790 | | p=0.329 | | p=0.138 | |
| **Smoker** | **never/ex** | 162 (70%) | 161 (61%) | 152 (61%) | 171 (69%) | 225 (64%) | 98 (68%) | 179 (71%) | 144 (59%) | 165 (65%) | 158 (65%) | 239 (67%) | 84 (61%) |
|  | **active** | 68 (30%) | 105 (39%) | 97 (39%) | 76 (39%) | 127 (36%) | 46 (32%) | 73 (29%) | 100 (41%) | 87 (35%) | 86 (35%) | 120 (33%) | 53 (39%) |
|  |  | p=0.021 | | p=0.056 | | p=0.380 | | **p=0.006** | | p=0.925 | | p=0.293 | |

Supplementary Table 2 Correlation analysis for associations between protein expression of YY1, CP2 and the YY1CP2-score and clinicopathological features. Correlations were analyzed using Fisher’s exact test or Chi-squared test. HPVhr, HPV high risk

|  |  | **Protein expression (n=102)** | | | | | |
| --- | --- | --- | --- | --- | --- | --- | --- |
|  |  | **YY1** | | **CP2** | | **YY1CP2** | |
|  |  | **low** | **high** | **low** | **high** | **negative** | **positive** |
| **T Stage** | **T1-2** | 56 (67%) | 17 (89%) | 41 (73%) | 32 (71%) | 58 (69%) | 15 (88%) |
|  | **T3-4** | 27 (32%) | 2 (10%) | 15 (26%) | 13 (28%) | 26 (30%) | 2 (11%) |
|  |  | p=0.055 | | p=0.814 | | p=0.142 | |
| **N Stage** | **N0** | 7 (8%) | 0 (0%) | 5 (8%) | 2 (4%) | 7 (8%) | 0 (0%) |
|  | **N1-3** | 76 (91%) | 19 (10%) | 51 (91%) | 43 (95%) | 77 (91%) | 17 (10%) |
|  |  | p=0.343 | | p=0.457 | | p=0.598 | |
| **Staging** | **I-II** | 4 (4%) | 0 (0%) | 3 (5%) | 1 (2%) | 4 (4%) | 0 (0%) |
|  | **III-IV** | 79 (95%) | 19 (10%) | 53 (94%) | 44 (97%) | 80 (95%) | 17 (10%) |
|  |  | p=1.000 | | p=0.627 | | p=1.000 | |
| **HPVhr** | **negative** | 65 (80%) | 9 (50%) | 44 (83%) | 29 (64%) | 65 (89%) | 16 (64%) |
|  | **positive** | 16 (19%) | 9 (50%) | 9 (16%) | 16 (35%) | 8 (10%) | 9 (36%) |
|  |  | **p=0.014** | | p=0.036 | | **p=0.011** | |
| **Sex** | **female** | 17 (20%) | 6 (31%) | 14 (25%) | 9 (20%) | 18 (21%) | 5 (29%) |
|  | **male** | 66 (79%) | 13 (68%) | 42 (75%) | 36 (80%) | 66 (78%) | 12 (70%) |
|  |  | p=0.362 | | p=0.551 | | p=0.529 | |
| **Age** | **<60** | 34 (40%) | 7 (36%) | 25 (44%) | 16 (35%) | 34 (40%) | 7 (41%) |
|  | **≥60** | 49 (59%) | 12 (63%) | 31 (55%) | 29 (64%) | 50 (59%) | 10 (58%) |
|  |  | p=0.741 | | p=0.355 | | p=0.957 | |
| **Smoker** | **never/ex** | 31 (37%) | 9 (47%) | 20 (35%) | 20 (44%) | 33 (39%) | 7 (41%) |
|  | **active** | 52 (62%) | 10 (52%) | 36 (64%) | 25 (55%) | 51 (60%) | 10 (58%) |
|  |  | p=0.420 | | p=0.373 | | p=0.884 | |

Supplementary Table 3 Univariable analysis of DFS for mRNA, CNV and protein expression of YY1, CP2 and the YY1CP2-score. Multivariable analysis was not calculated. HR, hazard ratio; CI, confidence interval

|  | **Univariable** | | |
| --- | --- | --- | --- |
| **mRNA expression (n=510)** | **HR** | **95% CI** | **p value** |
| **YY1** | 1.20 | 0.90-1.59 | 0.208 |
| **CP2** | 1.00 | 0.76-1.33 | 0.992 |
| **YY1CP2-Score** | 1.21 | 0.89-1.64 | 0.216 |
| **Copy number variation (n=510)** | |  |  |
| **YY1** | 1.13 | 0.86-1.50 | 0.383 |
| **CP2** | 0.83 | 0.62-1.10 | 0.189 |
| **YY1CP2-Score** | 0.96 | 0.70-1.32 | 0.812 |
| **Protein expression (n=102)** |  |  |  |
| **YY1** | 0.61 | 0.24-1.58 | 0.309 |
| **CP2** | 0.76 | 0.39-1.51 | 0.437 |
| **YY1CP2-Score** | 0.50 | 0.18-1.43 | 0.196 |
